# Myths and methodologies: Optimising experimental rigour in heat adaptation research: Menstrual status classification and scheduling approaches

**DOI:** 10.1113/EP093344

**Published:** 2026-04-02

**Authors:** Jessica A. Mee, Tessa R. Flood

**Affiliations:** ^1^ School of Sport and Exercise Science University of Worcester Worcester UK; ^2^ Department of Psychological and Sport Sciences, School of Human and Social Sciences University of West London London UK

**Keywords:** eumenorrhoeic, female physiology, heat acclimation, hormonal contraceptive, naturally menstruating, ovulatory cycles, phase specific testing, thermoregulation

## Abstract

Women remain underrepresented in thermal physiology research, particularly within studies examining physiological adaptation to hot environments. Among the limited research that includes female participants, few studies have appropriately classified menstrual status of their participants or rigorously accounted for ovarian hormone influences. Both endogenous and exogenous ovarian hormones have been demonstrated to influence thermoregulatory responses. Failing to control for these factors can confound interpretation; observed adaptations may reflect hormonal variation rather than true physiological adaptation. This methodological review offers guidance on incorporating ovarian hormone considerations into mechanistic heat adaptation studies, emphasising the highest standards of research rigour. We recognise applied research and real‐world implementation may necessitate greater methodological flexibility. Part 1 presents a three‐tiered approach (bronze‐to‐gold tier) to accurately classify menstrual status in female participants, enabling scalable implementation from low‐cost, low‐burden methods to higher‐resource, high‐rigour approaches. Part 2 offers guidance for experimental scheduling, including the potential benefits of standardising testing within defined menstrual phases, to minimise the confounding effects of endogenous and exogenous ovarian hormones. Crucially, given biological variability, this section also highlights the importance of transparent, detailed and consistent documentation of the testing timing relative to participants menstrual cycle or hormonal contraceptive use, regardless of approach, to enhance interpretation and reproducibility. By highlighting key methodological considerations, this review aims to promote methodological consistency and enhance the rigour of studies including women in heat adaptation research. Implementing these recommendations will support more valid comparisons across studies, facilitate meta‐analyses and ultimately contribute to the development of evidence‐based heat adaptation guidance for women.

## Abstract (highlights)

- **What is the topic of this review?**
  This review provides methodological guidance for incorporating ovarian hormone considerations into heat adaptation studies, intended solely for research purposes and focused on achieving the highest standards of methodological rigour.
- **What advances does it highlight?**
  This methodological review offers practical recommendations for study design, including the importance of accurately classifying female participants depending on logistical, practical and resource constraint. In addition, the review provides recommendations for the selection of defined menstrual phases for experimental trials. By standardising classification and scheduling approaches, the guidelines support methodological rigour, improve reproducibility, and enable more reliable interpretation of heat adaptation responses in women.

## 1 INTRODUCTION

Despite growing recognition that females are consistently underrepresented in sports, health, and medical research (Cowley et al., 2021; Hutchins et al., 2021; Kuikman et al., 2023; Smith et al., 2022), a persistent gap remains, particularly in the field of thermal physiology. The perceived complexities, lack of education, and costs of tracking menstrual cycles have created a significant deterrent for researchers, leading to a widespread failure to (1) include females as study participants, and (2) when using females, properly account for the influence of ovarian hormones on thermal physiology. Only 13% of heat adaptation study participants are female, and 90% fail to report menstrual status (Kelly et al., 2024). Accounting for menstrual cycle phase and hormonal contraceptive use is essential, as changes in core temperature during luteal‐to‐follicular and active‐to‐non‐active pill testing (Stachenfeld et al., 2000) can mimic heat adaptation changes in core body temperature. Crucially, no study confirmed naturally menstruating females to have eumenorrhoeic cycles (Kelly et al., 2024); that is, 21–35‐day cycle length, confirmed ovulation, a correct hormonal profile from blood samples, confirmed for 2 months (Elliott‐Sale et al., 2021; Smith et al., 2022). Within this paper the term *confirmed ovulation* will be used to describe evidence of a luteinising hormone surge through urinary ovulation testing and is introduced here for clarity on terminology. For full details on the three‐tiered system of menstrual classification, we refer you to Elliott‐Sale et al. (2021) and Smith et al. (2022). The lack of robust methodological control in existing research limits our understanding of how females of varying menstrual states respond to heat adaptation.

### 1.1 Ovarian hormone profiles

**FIGURE 1:**
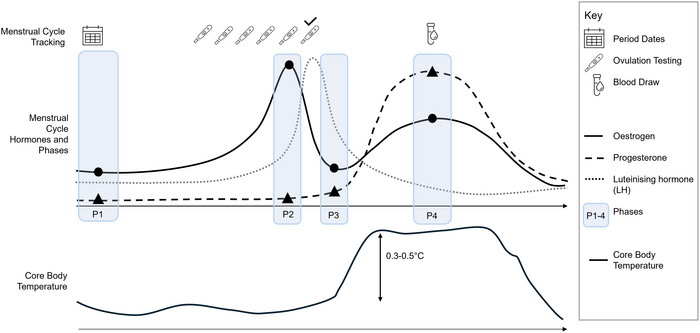
Visual representation of menstrual hormonal fluctuations in a normal cycle. Presented alongside the four menstrual cycle phases and menstrual cycle tracking protocol to determine eumenorrhoea. Also presented are typical core body temperature fluctuations across the menstrual cycle, shown as a visual representation of likely changes rather than as a recommended tracking method. Adapted from Elliott‐Sale et al. (2021) and Baker et al. (2020). P signifies phase: P1: menstruation (days 1–5) with low oestrogen and progesterone; P2: late follicular phase (14–26 h before ovulation), marked by elevated oestrogen and low progesterone (<6.36 nmol L^−1^); P3: 24–36 h after ovulation; P4: mid‐luteal phase typically occurring 7 days (range 6–8 days) after confirmed ovulation, when both oestrogen and progesterone are elevated (progesterone >16 nmol L^−1^).

The two most common ovarian hormone profiles studied within heat adaptation research are the menstrual cycle and hormonal contraceptive use (Kelly et al., 2024). A normal, healthy menstrual cycle is regulated by the hypothalamic–pituitary–ovarian axis and is characterised by large fluctuations in oestrogen and progesterone (Figure 1). It is defined by (1) regular 21‐ to 35‐day cycle, (2) confirmed ovulation, and (3) a progesterone rise ∼1 week after ovulation (Elliott‐Sale et al., 2021). Although often split into the follicular (before ovulation) and the luteal (after ovulation) phases, recent guidance recommends testing across four distinct phases (Elliott‐Sale et al., 2021, Figure 1). Hormonal contraceptives provide exogenous hormones that supress endogenous oestrogen and progesterone. They fall into three broad categories: oral contraceptive pills (OCPs), cyclic contraceptives, and long‐acting reversible contraceptives. OCPs include combined (oestrogen and progestin) monophasic (one continuous dosage) or combined phasic (two or more dosages) contraceptive pills and progestin‐only pills. Combined OCPs are typically taken with a scheduled hormone‐free break (e.g., 21‐day pill, 7‐day break), whereas progestin‐only pills are taken continuously. Cyclic contraceptives (combined) include the vaginal ring and contraceptive patch, also typically taken with a scheduled hormone‐free break. Long‐acting reversible contraceptives (progestin only) include the implant (lasts ∼3 years), hormonal coil (intrauterine system (IUS), lasts ∼3–5 years) and contraceptive injection (lasts ∼8–12 weeks), all used continuously. Within this paper we will use the UK definitions, whereby an IUS is a hormonal coil, and an intrauterine device (IUD or copper coil) is a non‐hormonal contraceptive and does not provide exogenous hormones. In the case of an IUD, users should therefore be classified as naturally menstruating. Because hormonal contraceptives vary widely across brands and countries, accurately identifying the specific type and brand used is essential.

### 1.2 Thermoregulation and ovarian hormone profiles

Endogenous and exogenous ovarian hormones have been demonstrated to influence thermoregulatory responses across both eumenorrhoeic cycles and oral contraceptive users. In eumenorrhoeic women, resting core temperature rises ∼0.3–0.5°C in the luteal phase with progesterone (Figure 1; Stachenfeld et al., 2000; Stephenson & Kolka, 1993), increasing the onset threshold for sweating and cutaneous vasodilation (Stachenfeld et al., 2000; Stephenson & Kolka, 1985). Effects on other heat adaptation markers are inconclusive; for more details we refer you to Baker et al. (2020) and Charkoudian and Stachenfeld (2014). In contraceptive users, thermoregulatory rhythms persist with an elevation in core temperature of ∼0.9°C and ∼0.4°C for progestin‐only and combined oral contraceptive users relative to the follicular phase, with changes also observed for sweating thresholds for progestin‐only users (∼0.7°C; Stachenfeld et al., 2000). However, more recent evidence has questioned the consistency and practical significance of menstrual cycle‐related temperature changes, suggesting that observed effects are small and variable and may not meaningfully alter thermoregulatory heat loss during exercise (Notley et al., 2019). Considering this variability, we maintain that any change in body temperature may confound heat adaptation outcomes and should be appropriately considered.

### 1.3 Heat adaptation

Chronic heat alleviation strategies induce heat adaptation through repeated exposures to hot environments, either naturally (heat acclimatisation), or in controlled settings (heat acclimation). Pre‐ and post‐experimental trials (sometimes referred to as heat stress or heat tolerance tests, and controlled metabolic heat production or performance trials) quantify adaptations that improve heat tolerance and reduce susceptibility to exertional heat illnesses (Périard et al., 2015), including, lower resting core temperature, an earlier onset of cutaneous vasodilation and sweating, and higher sweating rates (Taylor et al., 2014). Heat acclimation (HA) can be passive (e.g., saunas, steam rooms, water‐perfused suits, or hot water immersion), or active (e.g., exercise in the heat, e.g., fixed work rates, self‐selected work rates, controlled hyperthermia, clamped heart rate), or a combination (Gibson et al., 2019). Protocols vary in duration but ≥15 exposures (long‐term) are recommended to maximise adaptation (Périard et al., 2015; Saunders et al., 2019). Short‐term (e.g., 4–5 days) and medium‐term (e.g., 7–10 days) protocols have been developed to reduce time demands with 80% of adaptations occurring in 7 days (Robinson et al., 1943) though reductions in primary markers of HA including core body temperature are inconsistent (Tyler et al., 2016). Importantly, when assessing HA, the recommended four‐phase menstrual cycle testing approach becomes problematic, as even short‐ and medium‐term HA protocols inevitably span both the follicular and luteal phases. Therefore, applying four distinct testing phases is neither feasible nor methodologically appropriate for HA interventions.

### 1.4 Ovarian hormones and heat adaptation

In female participants, distinguishing true heat adaptation from ovarian hormone effects presents a significant challenge. HA lowers core temperature by 0.1–0.5°C (Lorenzo et al., 2010; Tyler et al., 2016; Zurawlew et al., 2018), whereas luteal phase and hormonal contraceptive use core temperature may rise by 0.3–0.7°C (Stachenfeld et al., 2000). If pre‐experimental trials occur in the luteal phase and post‐experimental trial in the follicular phase, or between active and non‐active pill phases, observed reductions may reflect hormonal shifts rather than adaptation. Hormonal changes also raise the temperature threshold for sweating and cutaneous vasodilation (Stephenson & Kolka, 1985) potentially shifting key adaptation markers. Therefore, it is essential that HA studies control for cycle phase and hormones to accurately interpret physiological adaptations caused by HA.

This review will present gold standard methodological considerations for female participants with a eumenorrhoeic menstrual cycle and who use hormonal contraceptives within HA research. Within the guidance presented below on profiling these groups, we will exclude participants who have a suspected menstrual dysfunction as their inclusion may introduce significant confounding variables that obscure the true effects of the intervention. This review will be presented in two parts,

1. Considerations for classifying the hormonal profiles of adult female participants (aged ≤40 years old) for thermal physiological research.
2. Considerations for the scheduling of HA and acclimatisation testing around the menstrual cycle and hormonal contraceptive use.

## 2 PART 1. CLASSIFYING MENSTRUAL STATUS FOR HEAT ADAPTATION RESEARCH

The details provided below are adapted from the most recent methodological guidance for including women in research and follow the three‐tier classification system to ensure transparency in reporting and appropriate HA scheduling (Elliott‐Sale et al., 2021; Smith et al., 2022). Briefly participants not using hormonal contraceptives will be defined as naturally menstruating for bronze classification, ovulatory for silver classification and eumenorrhoeic for gold classification which provides the highest level of methodological rigor. When discussing menstrual dysfunctions and/or abnormal uterine bleeding (AUB), this review will use the updated terminology from Oleka et al. (2024).

### 2.1 Bronze tier classification: Determining naturally menstruating participants

To ensure bronze classification, participants should be recruited who have a self‐reported ovarian hormone profile of naturally menstruating with cycle lengths of 21–35 days and no hormonal contraceptive use in the last 3 months. Tools like the ovarian hormone profile tool (Ovarian Hormone Classification Tool: Elliott‐Sale et al., 2024) can be used to ensure accurate self‐reporting. Participants using a non‐hormonal IUD (e.g., copper coil) should not be excluded at the self‐reported profile stage.

Once the self‐reported profile has been established, best practise recommends monitoring participants’ bleeding days every month throughout the study to calculate cycle lengths. At a minimum two cycles of calendar counting will help determine a naturally menstruating profile. If during prospective calendar counting, cycle lengths are consistently not 21–35 days, follow the below guidance.

- Cycles for 2 months shorter than 21 days. *Recommendation*: report that participant is not naturally menstruating and instead has suspected AUB‐frequent (polymenorrhoea).
- Cycles for 2 months longer than 35 days. *Recommendation*: report that participant is not naturally menstruating and instead suspected AUB‐infrequent (oligomenorrhoea).
- One cycle during tracking is shorter (<21 days) or longer (>35 days). *Recommendation*: report cycle lengths as variable and do not classify as naturally menstruating.

If utilising the bronze classification approach due to logistical, practical and resource constraints, participants must be referred to as naturally menstruating rather than eumenorrhoeic or ovulatory as these have not been confirmed with biological verification. Researchers must also state the limitations that it is possible that women who have AUB‐ovulation (anovulation) or AUB‐endometrial dysfunction (luteal phase deficiency) could be included in the study and that the fluctuations across the menstrual cycle are assumed and not verified.

### 2.2 Silver tier classification: Determining ovulatory participants

To ensure silver classification, researchers should follow the bronze guidance and in addition, use ovulation tests to verify the cycle is ovulatory; this provides a more robust verification of a participant's menstrual status. Ovulation tests are non‐invasive indirect predictors of ovulation and detect the luteinizing hormone surge which occurs prior to ovulation (Su et al., 2017). Participants should complete one ovulation test in the morning (1‐h window) starting approximately 5 days before the expected mid‐point of the cycle and continuing for 10 days or until a positive test is achieved, whichever comes first (e.g., in a cycle of approximately 28 days, ovulation testing should begin on day 9). Participants should provide photo evidence of tests taken to aid validity of the method (including date, time and positive/negative result). At a minimum ovulation testing should form part of tracking for one cycle prior to scheduling HA. If during tracking, challenges in ovulation testing occur, follow the below guidance.

- No positive ovulation test is detected in month 1 of tracking. *Recommendation*: continue to the next cycle and ensure that consistent timings are used. At least one month with positive ovulation tests are required before scheduling HA.
- No positive ovulation test is detected across two cycles of tracking. *Recommendation*: report the participant is not ovulatory and instead suspected AUB‐O (anovulation); do not group with ovulatory participants.

If ovulation is confirmed but hormones are not measured, participants should be classified as ovulatory. Without biochemical verification, researchers must note the possibility that women with possible AUB‐E (luteal phase deficiency) may be included in the sample.

### 2.3 Gold tier classification: Determining eumenorrhoeic participants

To ensure gold classification, participants should complete at least 2 months of prospective menstrual cycle tracking to verify a eumenorrhoeic menstrual cycle, in accordance with the three‐step method (De Jonge et al., 2019; Elliott‐Sale et al., 2021; Schaumberg et al., 2017). This includes adherence to the bronze and silver recommendations, with a blood sample taken to confirm Phase 4 progesterone concentrations, thereby achieving the highest level of methodological control. Once ovulation has been confirmed via a positive ovulation test, a blood sample should be collected 6–8 days later to assess sufficient serum progesterone concentration (Phase 4 blood draw, 16 nmol L^−1^). To ensure consistency, blood draw timing should be standardised to a 2‐h window within a participant. There is research to suggest that ovarian hormone concentrations can be impacted by exercise and caffeine, so typical standardisation should be in place (Bonen et al., 1979; Kotsopoulos et al., 2009; Schliep et al., 2012). Sufficient progesterone and confirmation of regular ovulatory cycles would indicate a eumenorrhoeic cycle. Researchers should note that due to limits in technology it is likely progesterone verification will occur retrospectively following the study completion.

If the concentration of progesterone is lower than the clinical cut off, we recommend researchers follow the below guidance.

**FIGURE 2:**
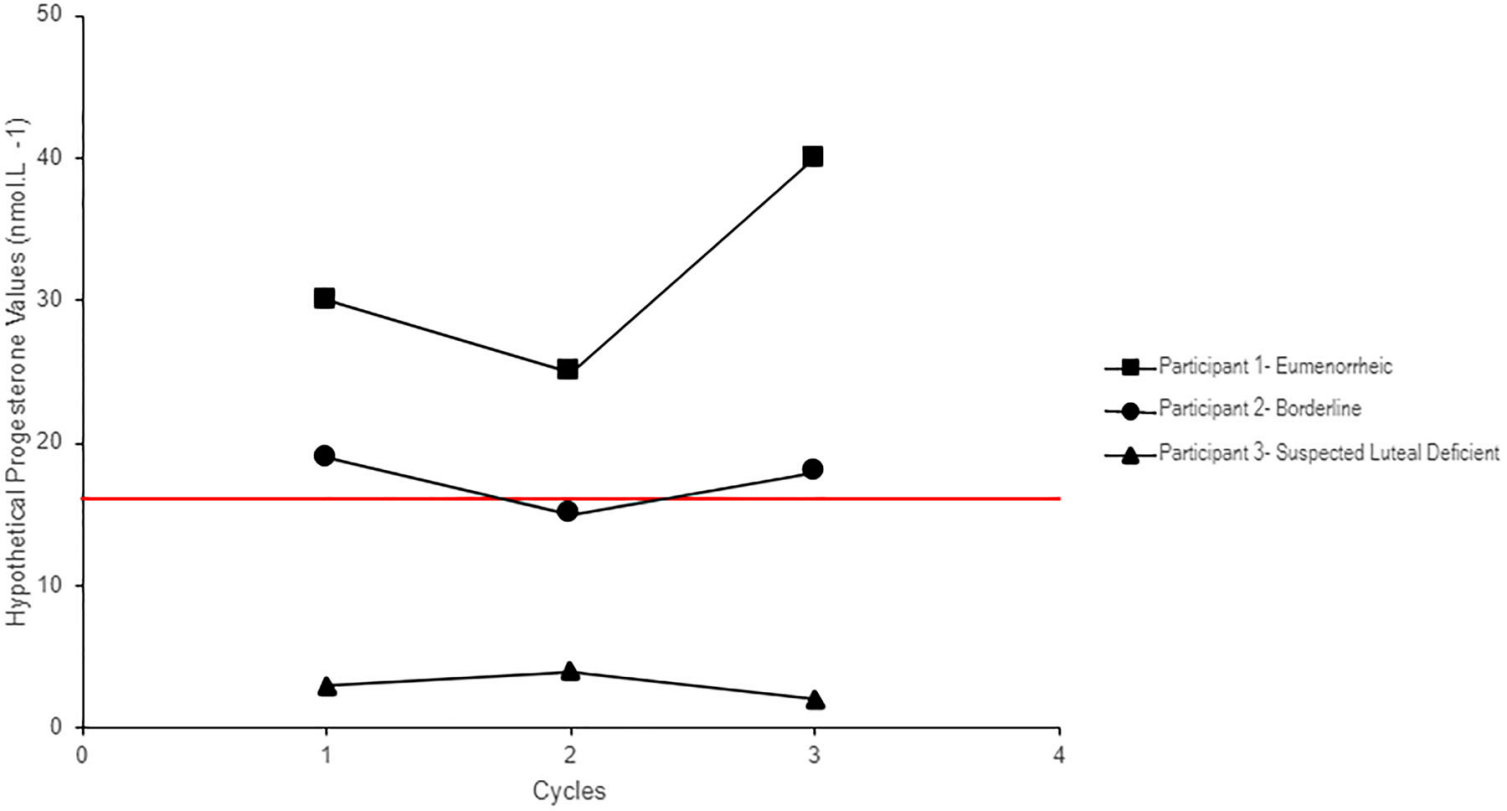
Visual representation of three participants’ ovarian hormone classification based on progesterone concentration and 3 months prospective tracking.

- In one sample, the progesterone concentration was slightly lower than the clinical cut off, but in the other samples the progesterone concentration was above the cut off. *Recommendation*: It is likely that the sample exhibiting lower progesterone concentrations was collected on an incorrect day, resulting in the progesterone peak being missed (Figure 2, participant 2). Assume this participant is eumenorrhoeic and continue using this participant.
- Progesterone concentration was below the cut off in all samples. *Recommendation*: report the participant is not eumenorrhoeic and instead suspected AUB‐E (luteal deficient cycle); do not group with eumenorrhoeic participants (Figure 2, participant 3).

Completing at least 2 months of prospective tracking to confirm eumenorrhoeic cycle will also provide information about the variability of the participants cycle which will aid the scheduling of HA in Part 2. To aid in this process, Supplementary File 1 contains a decision tree to assist researchers in completing this process.

### 2.4 Classifying hormonal contraceptive users

To ensure correct hormonal contraceptive classification, researchers must collect all details regarding the contraceptive used. Tools like the ovarian hormone profile tool (Ovarian Hormone Tool – Elliott‐Sale et al., 2024) can be used to aid transparency of reporting and ensure gold tier classification. At a minimum researchers must ensure participants have been using their current contraceptive for at least 3 months (detailing time on current contraceptive), hormonal contraceptive type, and brand (including dosage and hormones). If the hormonal contraceptive has a scheduled hormone‐free break (e.g., combined OCP), participants should determine whether this is being taken, as this would affect scheduling of HA. For gold tier classification researchers should look to include only one type of hormonal contraceptive per group, and in the cases of OCPs, group based on exogenous hormones and dosage (see Table 1 for more details; Smith et al., 2022). If it is not feasible to separate hormonal contraceptive types or to group OCPs according to their hormonal composition, then researchers should aim for silver tier, which requires detailed documentation of all characteristics within the group. We recognise the challenges this guidance presents, including the difficulty of recruitment and the potential exclusion of participants.

**TABLE 1:** Recent gold standard methodological guidance for grouping hormonal contraceptive users.

Methodological guidance	Rationale
One type of HC per group	Increase the homogeneity of the hormone profiles Different types of hormonal contraceptives have differences in exogenous hormones, delivery and timing
Group OCPs based on exogenous hormones and dosage	Increase the homogeneity of hormone profiles. Different OCPs have variations in exogenous hormone concentrations, progestin potency, androgenic and anti‐oestrogenic properties

HC, hormonal contraceptive; OCP, oral combined pill.

## 3 PART 2. SCHEDULING HA TESTING AROUND THE MENSTRUAL CYCLE AND HORMONAL CONTRACEPTIVE USE

In applied settings, HA protocols are often implemented with necessary flexibility. However, when the primary aim is to accurately quantify the magnitude of heat adaptation, greater control of menstrual cycle phase or hormonal contraceptive use becomes increasingly important. Accordingly, accurate classification of menstrual status, including naturally menstruating, ovulatory and eumenorrhoeic cycles under the bronze, silver and gold framework as well as hormonal contraceptive use outlined in Part 1, is essential for appropriate scheduling and interpretation of heat adaptation protocols. Despite this, current literature offers little guidance on how to schedule HA around menstrual cycle phase to support researchers in isolating true heat adaptations. This gap in understanding, poses challenges for designing HA studies with robust methodologies. Without clear recommendations, inconsistencies in testing schedules can confound results and limit the ability to draw robust conclusions about physiological adaptations in women. To address this limitation, we now offer practical guidance and examples on how future research may wish to schedule HA in consideration of menstrual cycle phases for eumenorrhoeic females and for HC users, thereby improving methodological consistency and enhancing the accuracy of reporting the magnitude of heat adaptations. To date, there is no empirical evidence confirming whether menstrual cycle phases influence the responses during a single HA session or the magnitude of adaptation; therefore, we have not specified where sessions should occur, but we advise maintaining consistency within participant groups to ensure comparability. More evidence is needed to indicate whether certain days of the menstrual cycle optimise responses to HA, and thus, inform scheduling more clearly.

### 3.1 Scheduling HA for naturally menstruating, ovulatory and eumenorrhoeic participants

To minimise the potential confounding influence of fluctuating ovarian hormones on HA markers, pre‐ and post‐experimental trials must be conducted during the same menstrual cycle phase. Researchers should select one of the four menstrual phases outlined previously in this paper and in accordance with Elliott‐Sale et al. (2021) (Figure 1). Testing during phase 2 or 3 is possible but not advised due to the narrow and difficult testing window. Instead, trials should be conducted in either (1) phase 1 or (2) phase 4; the appropriate phase will be determined by the classification approach described in Part 1.

Researchers should, where possible, schedule pre‐ and post‐experimental trials on the same menstrual cycle day across two consecutive cycles to ensure comparable ovarian hormone levels. If an interval of no testing occurs between the pre‐experimental trial and the start of HA, participants should maintain consistent activity levels, diet and sleep patterns, and avoid any additional interventions or heat exposures. Objective monitors such as activity trackers, sleep monitors or dietary logs are recommended to verify compliance.

#### 3.1.1 Approach 1: Testing in Phase 1 (during bleeding)

**FIGURE 3:**
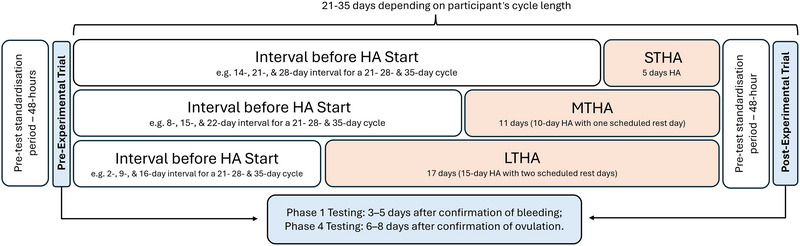
Example heat acclimation (HA) experimental schematics for short‐ (STHA), medium‐ (MTHA), and long‐term (LTHA) protocols in eumenorrhoeic females. Pre‐ and post‐experimental trials are scheduled during Phase 1 (3–5 days following confirmation of bleeding) or Phase 4 (6–8 days following confirmation of ovulation) of the menstrual cycle. The figure illustrate examples based on 21‐, 28‐ and 35‐day cycles; these do not represent all possible cycle lengths. Days shown reflect *count‐forward* days, not absolute menstrual cycle days.

Phase 1 offers stable and low concentrations of oestrogen and progesterone and is therefore ideal for participants who have been classified as naturally menstruating and ovulatory (bronze and silver tier). This approach is particularly appropriate when hormonal profiling is not available, as the onset of bleeding is easily identifiable and requires no additional costs. The pre‐experimental trial should occur 3–5 days after confirmed bleeding. HA then begins according to the duration of the protocol and whether conducted over consecutive or non‐consecutive days. Sufficient time should be allocated for a 48‐h standardisation period prior to the post‐experimental trial (see Figure 3). It is acknowledged that menstrual cycle length and timing can be variable, and that logistical, practical and resource constraints may occasionally prevent strict adherence to planned testing schedules. Therefore, while control of the experimental trial day is ideal for minimising biological variability, a degree of flexibility may be required, if deviations are clearly reported and considered during data interpretation. If bleeding differs from predicted timing, researchers should amend the day of the experimental trial and document the true cycle day and follow the guidance outlined below.

- If bleeding commences earlier or later than expected. *Recommendation*: Conduct the post‐experimental trial 3–5 days after confirmed bleeding, while still maintaining the 24–72‐h standardisation period between the final HA session and the post‐experimental trial.
- If bleeding is delayed (>72 h). *Recommendation*: Conduct the post‐experimental trial 72 h after the final HA session to accurately capture the full magnitude of heat adaptation and minimise potential decay of adaptations.
- If bleeding occurs >24 h earlier than anticipated. *Recommendation*: Complete the HA protocol, wait 24 h to allow adequate recovery and controlled standardisation period, then conduct the post‐experimental trial.

#### 3.1.2 Approach 2: Testing in Phase 4

Phase 4 experimental trials are also feasible when oestrogen and progesterone are high and stable. This approach is most appropriate when females have been classified as eumenorrhoeic (gold tier) with confirmed ovulation and hormone verification. Here, the pre‐experimental trial occurs 6–8 days after confirmed ovulation (e.g., day 20–22, if ovulation is on day 14). HA then begins according to the duration of the protocol and whether conducted over consecutive or non‐consecutive days. Sufficient time should be allocated for a 48‐h standardisation period prior to the post‐experimental trial (see Figure 3). If ovulation differs from predicted timings, researchers should amend the day of the experimental trial and document the true cycle day and follow the guidance outlined below.

- If ovulation testing is available during the HA intervention. *Recommendation*: test 6–8 days after confirmation of ovulation, with 24–72 h maintained between the final HA session and the post‐experimental trial.
- If ovulation testing is available but a positive test is not obtained. *Recommendation*: continue to test on the same day as the pre‐experimental trial.
- If ovulation testing is not available during the HA intervention. *Recommendation*: continue to test on the same day as the pre‐experimental trial.

As an example, a 15‐day HA protocol with rest days after days 5 and 10, in addition to a 48‐h standardisation period before the post‐experimental trial, would require a total of 19 days. Thus, within a 28‐day cycle, researchers should allow a 9‐day interval following the pre‐experimental trial before commencing the intervention (see Figure 3).

To ensure the highest quality research, for both Phase 1 and Phase 4 approaches, serum hormones should be verified on each experimental trial day to confirm pre‐ and post‐ hormonal comparability. Approach 2 (Phase 4) offers the advantage that progesterone >16 nmol L^−^^1^ verifies a eumenorrhoeic cycles in two menstrual cycles, as outlined in Part 1. If ovarian hormonal profiling cannot be completed or verified due to logistical, practical or resource constraints, or unplanned protocol deviations, strict consistency in menstrual phase and testing remains essential for methodological reliability. In such cases, testing should be conducted on the same cycle day or estimated menstrual cycle day, with detailed documentation of cycle day and phase determination methods, to account for expected biological variability.

### 3.2 Scheduling HA for hormonal contraceptive users

Testing guidelines for hormonal contraceptive users allow more flexible scheduling due to stable exogenous and endogenous hormone levels. Where possible, serum hormones should be verified on each experimental trial day to confirm consistency.

Where possible, it is important to use participant groups consistent in their hormonal contraceptive type to minimise variability and enhance study validity. However, we recognise that this approach can present substantial recruitment and logistical challenges, often making studies large and difficult to manage, especially, when attempting to include all contraceptive types. As such, if mixed contraceptive types are included within a single participant cohort, it is crucial to clearly identify each participant's contraceptive type within the dataset. We also recommend that all contraceptive details are clearly reported in any published paper to facilitate accurate interpretations by readers.

From Part 1, researchers should have established key details of participant's hormonal contraceptive use. Guidelines for scheduling HA differ slightly depending on whether participants use (1) continuous hormonal contraceptives, or (2) hormonal contraceptives with scheduled hormone‐free intervals.

#### 3.2.1 Continuous hormonal contraceptive use

**FIGURE 4:**
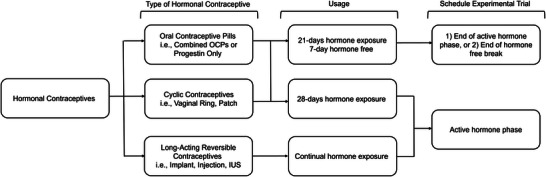
Scheduling of experimental trials before and after HA based on hormonal contraceptive type that either provide a scheduled hormone‐free period or that a provide continual hormone exposure. Oral contraceptive pills (OCP) include monophasic combined pill (e.g., Microgynon®, Yasmine®, Rigevidon®, Levest® and Lucette®), phasic (bi or tri) combined pill (e.g., Logynon®), and progestogen‐only pill (e.g., Norgeston®, Cerazette®). Cyclic contraceptives include vaginal ring (e.g., NuvaRing®) and patch (e.g., Evra®). Long‐acting reversible contraceptives include implant (e.g., Nexplanon®), injection (e.g., Depo‐Provera®, Noristerat®) and intrauterine system (IUS) (e.g., Mirena®, Jaydees®, Levosert® and Kyleena®).

Pre‐ and post‐experimental trials can occur anytime during the active hormone phase (see Figure 4). Long‐acting reversible contraceptive users should avoid any method changes, for example, if a device replacement is due, testing should be postponed until after insertion.

#### 3.2.2 Hormonal contraceptive use with scheduled hormone‐free intervals

For participants using hormonal contraceptives that include a scheduled hormone‐free interval the pre‐ and post‐experimental may be scheduled either toward the end of the active hormone phase or near the end of the hormone‐free interval, depending on the study design. To maintain consistency, testing should occur on the same day within two consecutive 28‐day contraceptive cycles. If participants have previously alternated between scheduled breaks and continuous use, they must have maintained a consistent usage pattern (e.g., continuous use without breaks) for at least 3 months prior to testing

## 4 CONSIDERATIONS FOR IMPLEMENTATION

We acknowledge that rigorous testing of eumenorrhoeic participants may be limited by logistical, practical and resource constraints. Researchers should report female participants using the three‐tiered classification framework (bronze–gold) and ensure accurate use of the terms naturally menstruating, ovulatory or eumenorrhoeic, and avoid using without appropriate verification. To support implementation, we provide guidance on associated costs, and practical considerations for researchers and applied settings.

### 4.1 Costing

Within this review we advocate for the use of at least 2 months of prospective menstrual cycle tracking before scheduling HA. For clarity, we provide approximate costs for eight participants completing this tracking. Additional tracking during HA to confirm ovulation and ovarian hormone concentrations may be required, depending on experimental design. Table 2 details the approximate cost for two boxes of Clearblue digital ovulation tests per participant. Clearblue tests are considered the highest quality option, which provide computerised results and reduce interpretation error compared with cheaper, visually interpreted tests (Schmalenberger et al., 2021). In addition, we provide costings for enzyme‐linked immunosorbent assay (ELISA) plate, sufficient to analyse two samples for eight participants in duplicate.

**TABLE 2:** Estimated 2‐month menstrual tracking costs for an eight‐participant cohort.

	Mean	Range [lowest–highest]
Ovulation testing^a^		
1 box (10 tests)	£23	£18–31
8 participants (2 boxes each)	£374	£282–504
Progesterone verification^b^		
1 ELISA plate	£438	£228–600
SUM	£813	£510–1104

^a^ Approximate cost for ovulation testing is determined from average cost of Clearblue digital ovulation tests from eight online retailers shipping to the UK (searched October 2025). Prices may vary overtime and between countries. Cheaper alternatives for ovulation tests are available.

^b^ Approximate cost for progesterone verification was determined from the average cost of progesterone ELISAs from seven competitive brands (cost verified November 2025). Alternative brands are available.

The most recent methodological recommendations suggest that serum blood sampling is the gold standard method for verifying ovarian hormone concentrations. Saliva sampling is a non‐invasive alternative; however, there are no equivalent hormone cut‐offs for menstrual cycle phases. Current recommendations suggest a sample should be collected in Phase 1 and then a second in Phase 4. When analysed progesterone concentration should be 1.5 times greater than Phase 1 and at least 50 pg mL^−1^ (Ferrer et al., 2024).

### 4.2 Individualisation of HA scheduling

HA protocols must be individualised to each participant's unique cycle length, bleeding onset and ovulation timing. The variability in cycle duration and hormones necessitates tailored modifications to the HA schedule. While we provide example frameworks based on common 21‐, 28‐ and 35‐day cycles, these are not exhaustive or universally applicable. Researchers should adapt protocols flexibly to accommodate individual menstrual patterns, ensuring that pre‐ and post‐experimental trials align with relevant cycle phases to capture accurate physiological heat adaptations. Completing prospective tracking prior to experimental trials will help aid individualisation of scheduling. Additionally, when studies aim to assess adaptation decay or retention, further considerations are required.

### 4.3 Considerations for applied research and practical constraints

While the guidelines presented in this review are designed to support mechanistic research aimed at understanding the physiological underpinnings of heat adaptation in females, we recognise that applied research or practice often operates under different constraints. In real‐world settings, such as athletic training, occupational heat exposure or military deployment, the timing and duration of HA protocols are often dictated by schedules, performance demands or operational requirements. Consequently, strict alignment with menstrual cycle phases or hormonal contraceptive use may not be feasible. Importantly, to date there is no evidence that scheduling HA in a particular phase of the menstrual cycle or contraceptive use confers superior adaptations. Thus, unless the objective is to precisely quantify the magnitude of adaptations for mechanistic understanding, modifying schedules and timing of the pre‐ and post‐experimental trials for this purpose is not necessary. These guidelines are not intended to discourage or restrict applied research or implementation of HA in an applied setting. Rather, they provide a framework to help researchers accurately measure adaptation when investigating mechanistic questions. Applied studies can still generate valuable insights and meaningful outcomes even when menstrual cycle or hormonal contraceptive phase considerations are not strictly controlled, though clear and detailed documentation of the known menstrual cycle characteristics and testing days remains important for interpretation.

## 5 CONCLUSION

This review offers guidance for conducting rigorous heat adaptation research in women, with a primary focus on well‐controlled mechanistic laboratory studies, while also considering flexibility for translation into applied settings. Menstrual status should be confirmed prior to scheduling HA using the bronze, silver or gold tier classification, with any associated limitations being discussed as appropriate. Pre‐ and post‐experimental trials should be scheduled with comparable hormone levels to ensure responses reflect true adaptation. Recommendations we have provided are deliberately non‐prescriptive, providing scientifically grounded guidance to enhance methodological consistency, reproducibility and robust interpretation of heat adaptation responses in women.

## AUTHOR CONTRIBUTIONS

Jessica Mee and Tessa Flood conceptualised the manuscript. Tessa Flood wrote part 1, Jessica Mee wrote part 2, and both reviewed, edited and co‐produced the final manuscript, figures and tables. Both authors have read and approved the final version of this manuscript and agree to be accountable for all aspects of the work in ensuring that questions related to the accuracy or integrity of any part of the work are appropriately investigated and resolved. All persons designated as authors qualify for authorship, and all those who qualify for authorship are listed.

## CONFLICT OF INTEREST

None declared

## Supporting information



Supplementary File 1. Menstrual cycle tracking decision tool.
