# Supplementary figures and images for: Myths and methodologies: Optimising experimental rigour in heat adaptation research: Menstrual status classification and scheduling approaches
Source: Exp Physiol. 2026 Apr 2:10.1113/EP093344. Online ahead of print. doi: 10.1113/EP093344 (PMC13394271; doi:10.1113/EP093344)

Prior 3 months cycles (21-35 days)

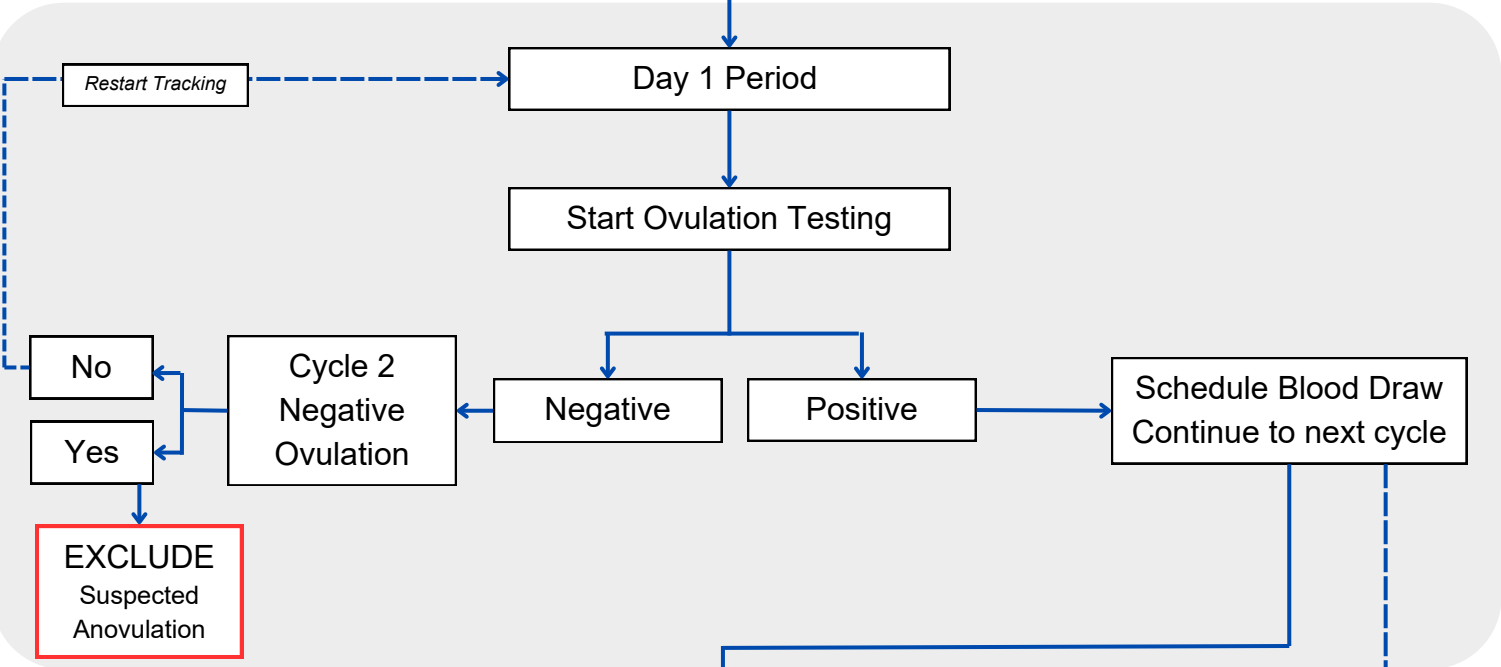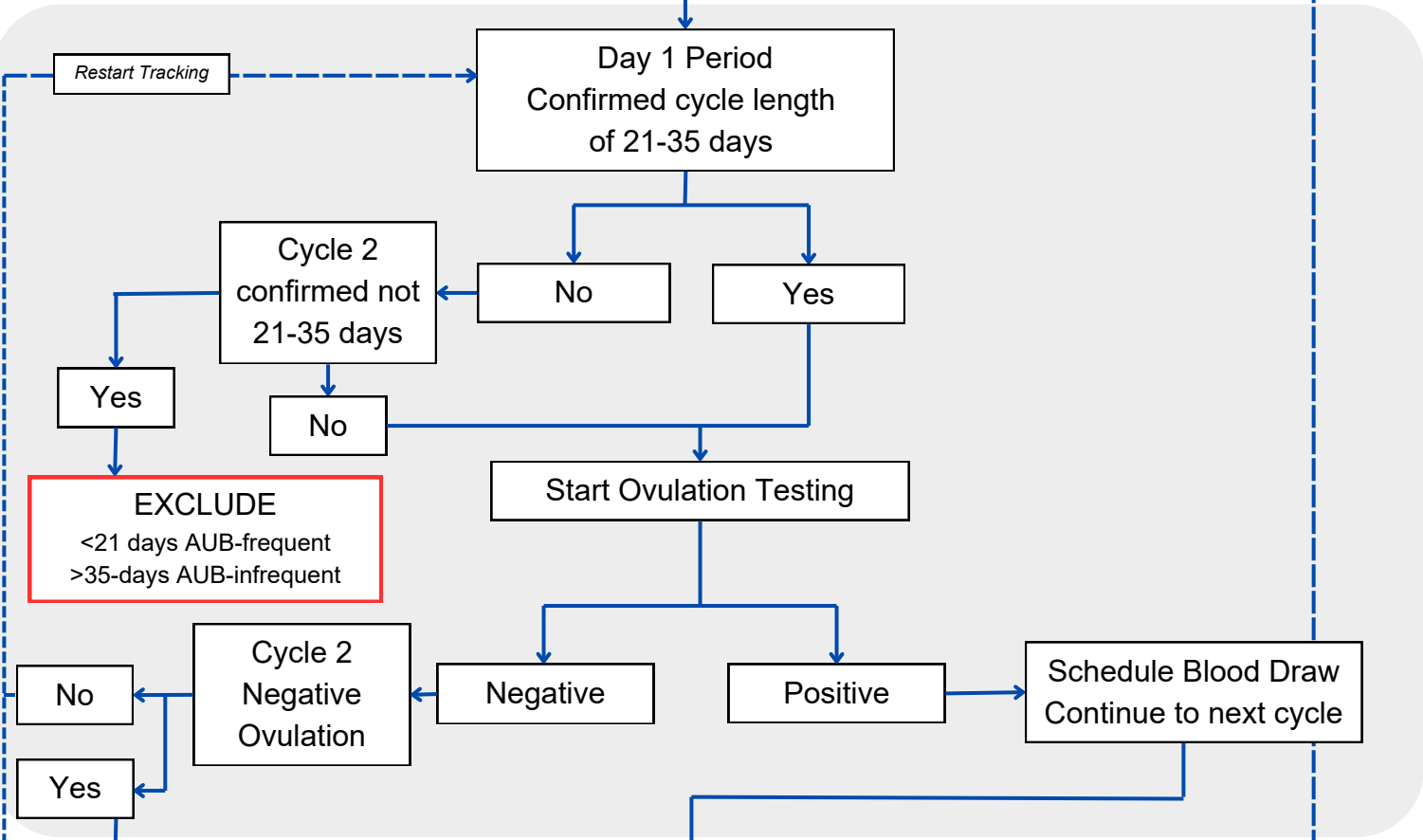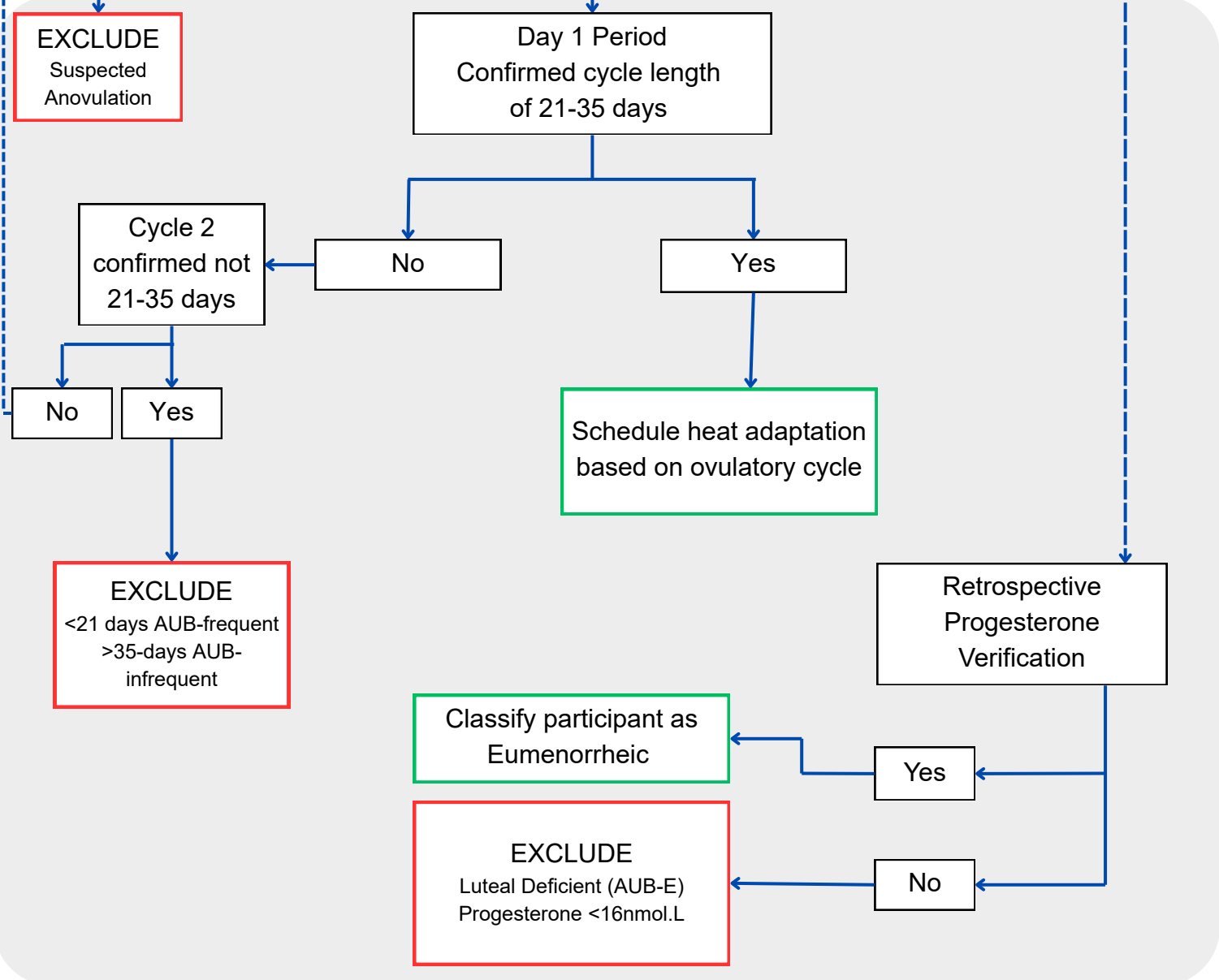

Supplement: Supplementary file 1 — Menstrual cycle tracking decision tool. [file EPH-9999-0-s001.pdf]
